# Implications of the Matrix Metalloproteinases, Their Tissue Inhibitors and Some Other Inflammatory Mediators Expression Levels in Children Obesity-Related Phenotypes

**DOI:** 10.3390/jpm14030317

**Published:** 2024-03-19

**Authors:** Aldona Wierzbicka-Rucińska, Izabela Kubiszewska, Renata Grzywa-Czuba, Lidia Gackowska, Mieczysław Szalecki, Jacek Michałkiewicz, Joanna Beata Trojanek

**Affiliations:** 1 Department of Biochemistry & Experimental Medicine, The Children’s Memorial Health Institute, Al. Dzieci Polskich 20, 04-730 Warsaw, Poland; 2 Department of Immunology, Nicolaus Copernicus University Collegium Medicum, M. Sklodowskiej-Curie 12 9, 85-094 Bydgoszcz, Poland; i.kubiszewska@cm.umk.pl (I.K.);; 3 Department of Microbiology and Clinical Immunology, The Children’s Memorial Health Institute, Al. Dzieci Polskich 20, 04-730 Warsaw, Poland; r.grzywa@ipczd.pl (R.G.-C.); jjmichalkiewicz@wp.pl (J.M.); j.trojanek@ipczd.pl (J.B.T.); 4 Department of Endocrinology and Diabetology, The Children’s Memorial Health Institute, 04-730 Warsaw, Poland; m.szalecki@ipczd.pl

**Keywords:** MMP-9, TIMP-1, IL-1 α, IL-6, TNFα, leptin and resistin

## Abstract

Objectives: Matrix metalloproteinases (MMPs) are calcium-dependent zinc-containing endo-peptidases engaged in many biological processes including adipogenesis, angiogenesis, and tissue remodeling. Fat tissue infiltration by peripheral leukocytes plays an important role in transition of fat tissue residual, non-inflammatory status into the pro-inflammatory one, resulting in fat tissue inflammation and expansion as well as production of many mediators like adipokines and cytokines. The aim of this study was to investigate the expression of MMPs, their endogenous tissue inhibitors (TIMPs), and selected inflammatory mediators in leukocytes and plasma of children with simple obesity to find their associations with obesity-related phenotypes. Material and methods: Twenty-six overweight/obese children and twenty-three healthy volunteers participated in the study. The leukocyte mRNA expression levels of *MMP-2*, *-9*, *-12 -14*, *TIMP-1*, *-2*, and IL*-6* were analyzed by the real time quantitative PCR. Plasma MMP-9/TIMP-1 and MMP-2/TIMP-2 ratios as well as the concentrations of MMP-9, TIMP-1, IL-1 beta, IL-6, TNF- alpha, leptin and resistin were tested by ELISA assays. Gelatin zymography was used to assess the activity of the leukocyte MMPs proteins. Results: The obese children showed the following: a) increased expression of leukocyte *TIMP-1* and slight elevation (close to statistical significance) of leukocyte *MMP-9* (*p* = 0.054), the decline in *MMP-2*, b) elevation of plasma MMP-9, leptin, and MMP9/TIMP1 ratio, c) reduced expression of plasma TNF-alpha and MMP-2/TIMP-2 ratio. Several negative correlations were found: *TIMP2* vs. ALT (r = −0.536), AST (r = −0.645) and TTG (r = −0.438), *IL-6* vs. GGTP (r = −0.815), and *MMP12* vs. TTG (r = −0.488), leptin vs. ALT (r = −0.569), MMP-9 vs. total cholesterol (r = −0.556). The only positive correlation was that of plasma leptin level vs. GGTP (r = 0.964). Conclusions: At the beginning of obesity development (children), possibly compensatory reactions prevail, reflected here by an increase in the expression of leukocyte MMPs inhibitor *TIMP-1*, decrease in the level of leukocyte *MMP-2* and plasma MMP-2, MMP2/TIMP-2 ratio, low plasma TNF-alpha and negative correlations between the expression of *TIMP-2* and liver (AST, ALT) or fat (TTG) inflammatory markers.

## 1. Introduction

According to WHO (the World Health Organization) regulations, overweight affects about 17% of children and adolescents aged 11–15, with a predominance of boys over girls (25% and 18% respectively), aged 11–12 [1]. Like in adults, childhood obesity is a multisystem disease that may lead to serious health problems, such as hypertension, metabolic syndrome, asthma, precocious puberty as well as insulin resistance and diabetes mellitus type II [2]. Obesity is a highly pro-inflammatory process that develop due to massive fat tissue expansion (adipocyte hypertrophy and hyperplasia) that leads to hypoxia and oxidative stress. It results in the transition of anti-inflammatory fat tissue status to pro-inflammatory one due to the transition of residual, anti-inflammatory macrophages (M2 phenotype) into pro-inflammatory one (M1 phenotype). This process mainly depends on the massive infiltration of fat tissue by peripheral immune cells (monocytes, lymphocytes, neutrophils) as well as the presence of numerous inflammatory mediators, including adipokines, cytokines, fatty acids, and lipids [3,4]. Fat tissue expansion is associated with systemic low grade inflammation which induces acute phase response due to the action of plasma pro-inflammatory cytokines (TNF-alpha, IL-6, IL-1beta) released by fat macrophages and other cells. They stimulate hepatocytes for acute phase protein production and mobilize bone marrow for increased production of an array of immune cells that enter the circulation. At least part of these cells, including monocytes, T cells, B cells, granulocytes, and mast cell precursors may infiltrate the fat tissue and participate in their expansion and transition into inflammatory phenotype. Accordingly, we want to assess the profile of *MMPs* and *TIMPs* gene expression (*MMP-2*, *9*, *12*, *14*, *TIMP-1,2*) in peripheral leukocytes, and plasma levels of MMP-2, 9, TIMP-1,2 of children with simple obesity to find out if they correlate with obese-related parameters [5,6,7]. MMP-2 and MMP-9 belong to a family of gelatinases which are able to degrade denatured collagens/gelatin, laminin. MMP-2 (not MMP-9) can cleave fibrillary collagen, both can target non-fibrillary collagens type IV, V, VII and X as well as elastin and fibronectin [8]. Both MMP-9 and MMP-2 are expressed by monocytes, neutrophils and lymphocytes and participate in immune reactions by modulation of cytokines and chemokines receptors expression in their target cells. MMP-2 and MMP-9 are engaged in adipose tissue growth differentiation and expansion by ECM components degradation. Fat tissue can produce and secrete active MMP-2 and MMP-9 [9]. Their expression increases during adipocyte differentiation and fat tissue expansion while their inhibition markedly reduce adipocyte differentiation. Obese subjects express increased levels of circulating MMP-2 and MMP-9 [10]. Fat tissue growth and differentiation is associated with high production of collagen type IV by mesenchymal stem cells. Simultaneously, the cells develop enzymatic capabilities to remodel basement membrane, via activation of MMP-2 and MMP-9, with complex regulation by MT1-MMP (MMP-14) that facilitates the activation of proMMP-2 by interacting with TIMP-2. It acts as a bridge between proMMP-2 and MMP-14 and promotes transition of proMMP-2 into active MMP-2. MMP-14 participates in nutritional stress and obesity development by its engagement in adipose tissue remodeling and metabolism [11,12]. MMP-12 exhibits high specificity for elastin degradation and is produced mainly by activated macrophages. In humans, MMP-12+ macrophages signature in the white adipose tissue (WAT) was associated with inflammation and insulin resistance [13]. On the other hand, MMP-12 expressing macrophages presented M2 anti-inflammatory phenotype which might serve as a compensatory mechanism to restrain adipose tissue expansion and insulin resistance [14]. Tissue inhibitors of MMP (TIMPs 1–4) are proteins present in the extracellular matrix in soluble forms, except for TIMP3 which binds to the ECM [15], TIMPs reversibly regulate MMPs activities by specific binding and forming 1:1 stoichiometric complex. TIMP-1 and TIMP-2 plasma levels are increased in patients with metabolic syndrome and their levels correlate with obesity indices including BMI, waist circumference and metabolic parameters [16]. The elevation of TIMP-1 and TIMP-2 follows that of MMP-2 and MMP-9 possibly to limit the activity of MMPs that could be deleterious [17]. So far there are no studies on the connection between leukocyte and plasma expression of MMPs and TIMPs with inflammatory markers of obesity including circulating levels of resistin, AST, ALT and TNF-alpha. Here we want to find out whether the expression of these inflammatory markers will be changed and associated with simple obesity-related phenotypes in children.

## 2. Materials and Methods

The study was conducted in accordance with the Declaration of Helsinki and was approved by the Ethics Committee of the Children’s Memorial Health Institute (decision no 162/KBE/2011). All parents and children aged >12 years provided written informed consent. The research was conducted on pediatric patients—boys aged 10–16 years (mean age 13.1 ± 2.9 years). The limitation of the study was the participation of only boys and no girls (p. 13). The following groups were included: 26 patients with simple obesity diagnosed in the Department of Endocrinology and Diabetology of the Children’s Memorial Health Institute in Warsaw and 23 healthy, slim children in the control group. Basic anthropometric measurements (weight and height to determine body mass index BMI) were performed in all patients and healthy adolescents of the control group. The blood samples were collected at the baseline between 7:00 and 9:00 am, after 12 h of fasting. Blood samples were centrifuged at 4000 rpm for 10 min and the isolated sera were stored at −80 °C until biochemical analysis. Serum concentrations of alanine aminotransferase (ALT), aspartate aminotransferase (AST), glucose, gamma-glutamyltransferase (GGTP), total cholesterol (TC), triglyceride (TTG), low-density lipoprotein (LDL-c), high-density lipoprotein (HDL-c), and high sensitivity C-reactive protein (hsCRP) were assessed using Cobas 501 autoanalyzer (Roche) (Table 1).

### 2.1. RNA Isolation and Real Time PCR Technique

Leukocytes were isolated from venous blood by Histopaque gradient centrifugation (Sigma-Aldrich 119, St. Louis, MI, USA). Total RNA was isolated by Chomczyński method using Trizol Reagent (Invitrogen, Waltham, MA, USA). Using absorbance at 260 nm and 280 nm, the RNA concentration in each sample was determined, and the purity/integrity was checked. One microgram of total RNA per sample was converted to cDNA in reverse transcription polymerase chain reaction (RT-PCR) using Taq Man reverse transcription reagents. Quantitative RT-PCR (Q-PCR, real-time PCR) for the following target genes: MMP-2, MMP-9, MMP-12, MMP-14, TIMP-1, TIMP-2, IL-6 and the endogenous control (reference gene) glyceraldehydes-3-phosphate dehydrogenase (G3PDH) were performed in the Via 7 Real Time System, according to manufacturer’s recommendation. For one reaction 50 ng of cDNA was loaded with SYBR Green PCR Master Mix, and 10 nmol/L for each of the forward and reverse primers (Table 2). The specificity of the amplification reaction was verified by the melting curve analysis. The relative fold changes in target gene expression between obese patients and the control leukocytes were determined by normalization to the expression of the reference gene G3PDH, by using Pfaffl’s mathematical model [18], as previously described [19]. All reagents, equipment, and other materials used in the Q-PCR technique were provided by Thermo Fisher Scientific (Waltham, MA, USA).

### 2.2. MMP Activity Assay

Total peripheral leukocytes isolated by venous blood gradient centrifugation was cultured in RPMI medium without FBS with the addition of 1 µg/mL LPS in the time range: 0′, 10′, 30′, 1 h, 4 h, 10 h, 24 h, and 72 h [20,21]. Gelatin zymography for MMP activity was performed according to previously published methods (by other authors) [22,23,24]. At the specified times, the leukocyte culture supernatants were collected, and then 25 µg of protein (measured by Nanodrop) was applied to 8% polyacrylamide gel containing 2 mg/mL gelatin under reduced conditions. After electrophoresis, the gels were washed (2 × 20 min) with 2.5% Triton X-100 to remove SDS (Renaturing buffer) and placed for incubation at 37 °C in Developing buffer consisting of 50 mM Tris/HCl pH 7.5, 150 mM NaCl, and 5 mM CaCl_2_. After at least 16 h of incubation, the gels were first stained with 0.05% Coomassie brilliant blue G-250 in a solution of 2.5:1:6.5 methanol/acetic acid/water and then destained for 1 h in 20% isopropanol with 10% acetic acid.

### 2.3. ELISA Method

Leptin (DRG International, Inc. Springfield, NJ, USA) and resistin (Phoenix Pharmaceuticals, Inc., Burlingame, CA, USA) ELISA kits contained pre-coated ELISA plates. The assays were performed according to manufacturer’s instructions. ELISA kits for MMP-9, TIMP-1, MMP9/TIMP1 and MMP2/TIMP2 were obtained from R&D Systems Inc., Lehi, UT, USA (DuoSets) but IL-1β, IL-6 and TNFα sets were obtained from BD Biosciences, Waltham, MA, USA (OptEIA Sets). The amount of measured protein in the sample was calculated from a reference curve generated in the same assay with reference standards with known concentrations of appropriate protein. Standard curves for the various proteins were constructed using a four-parameter regression formula and plotted as a linear curve (log-log). Cytokine concentrations in experimental samples were calculated using Genesis software, version 2.2. Results are expressed in picograms or nanograms per mililiter. In assays, in which plasma probe or leukocyte culture supernatants were diluted before testing, the results were multiplied accordingly.

### 2.4. Statistical Analysis

In order to perform statistical analysis for various groups of variables, the compliance with the normal distribution was tested (W Shapiro-Wilk test), and an appropriate statistical test: a parametric (t-student) or non-parametric (U-Mann-Whitney) was selected. Nonparametric tests were used to estimate differences between both study groups. Spearman’s rank correlation coefficient was used to determine the relationship between variables (clinical, anthropometric, biochemical, molecular and immunological parameters). All demonstrated differences and specific correlations indices were considered statistically significant at the *p* < 0.05 level. The statistical program 12 Stat Soft was used for all the tests and analysis.

## 3. Results

**Figure 1:**
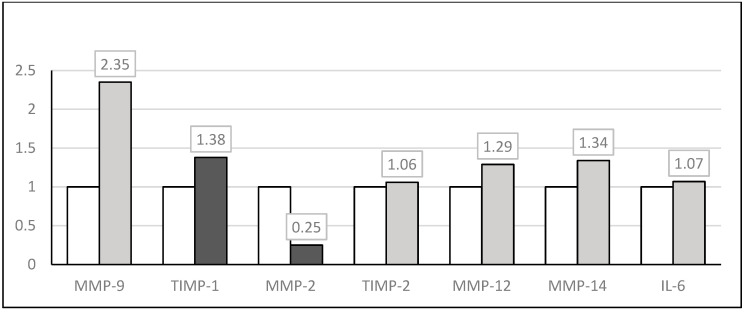
The expression of MMPs, TIMPs and IL-6 in obese children and the control group. Statistical significant value (≤0.05) marked as black color, non-significant—gray color, control—white color.

Leukocytes from obese children showed significantly higher expression of *TIMP-1* (*p* = 0.048). The MMP-9 expression tended to be elevated (*p* = 0.054), as compared to the leukocytes of the control group. *MMP-2* expression (*p* = 0.002) was slightly, but significantly decreased. Expression of the remaining genes tested (*TIMP-2; MMP-12; MMP-14* and *IL-6*) remained unchanged (Table 3, Figure 1). We also observed some significant negative correlations between leukocyte gene expression patterns and serum, liver, or adipose tissue metabolic parameters: leukocyte *TIMP2* level was negatively correlated with ALT (r = −0.536), AST (r = −0.645) and TTG (r = −0.438); leukocyte *IL-6* expression was negatively correlated with GGTP (r = −0.815), and leukocyte *MMP12* was negatively correlated with TTG (r = −0.488).

The average concentration of the following parameters: MMP-9, leptin and MMP9/TIMP1 ratio were significantly higher in obese study group compared to the control group. In the obese study group, compared to the control group, the TNF-alpha concentration and MMP-2/TIMP-2 ratio decreased significantly (Table 4). Reversible correlations were also found between mRNA expression in leukocytes and the concentration of metalloproteinases, their tissue inhibitors, cytokines and adipokines in plasma and metabolic parameters in serum, such as: *TIMP2* expression correlated with ALT (r = −0.536), AST (r = −0.645), TTG (r = −0.438); *IL-6* with GGTP (r = −0.815); *MMP12* with TTG (r = −0.488); leptin level correlated with ALT (r = −0.569), and MMP-9 protein concentration correlated with total cholesterol (r = −0.556). Only one positively correlation was the level of leptin in plasma with GGTP (r = 0.964).

**Figure 2:**
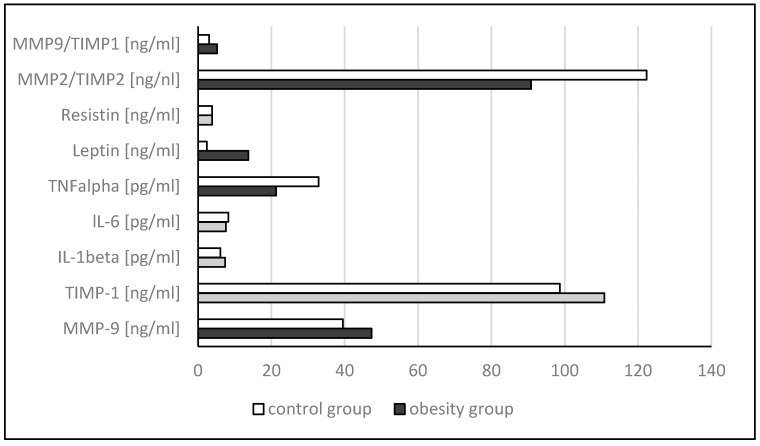
The concentration of MMPs, TIMPs, cytokines and adipokines in plasma in obese children and the control group. Statistical significant value (≤0.05) marked as black color, non-significant—gray color, control—white color.

The average concentration of the following parameters: MMP-9, leptin, and MMP9/TIMP1 ratio were significantly higher in the obese study group compared to the control group. In the obese study group, the TNF-alpha concentration and MMP-2/TIMP-2 ratio decreased significantly (Table 4, Figure 2). There were significant correlations between leukocyte mRNA gene expression and plasma levels of MMPs and TIMPs as well as serum concentrations of leptin, TNF-alpha, ALT, AST and TTG: *TIMP2* expression correlated with ALT (r = −0.536), AST (r = −0.645), TTG (r = −0.438); *IL-6* with GGTP (r = −0.815); *MMP12* with TTG (r = −0.488); leptin level correlated with ALT (r = −0.569), and MMP-9 protein concentration correlated with total cholesterol (r = −0.556). Only one positive correlation was that of the plasma leptin level with GGTP (r = 0.964).

**Figure 3:**
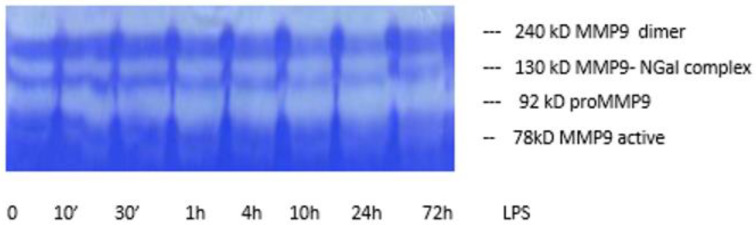
A represantive zymography from supernatant of obese patients leukocyte cultured with 1 μg/mL LPS.

As the result of zymography studies four bands were visible on the gels. These are in order from the top: MMP-9 dimer, MMP-9-N-Gal complex, proMMP-9, and MMP-9 active. The intensity of the bands increased with increasing exposure time to LPS in culture from 10 min to 24 h, and then decreased for 72 h (Figure 3).

## 4. Discussion

All together the findings presented here suggest that in the course of simple obesity in children, systemic inflammatory reactions may be rather limited. It was reflected here by decline in leukocyte *MMP2* expression, decreased serum MMP2 concentrations, low MMP2/TIMP2 ratio, and reduced serum TNF-alpha concentrations. Additionally, several negative correlations were found including: (a) leukocyte *TIMP2* expression versus serum liver (ALT, AST) and fat (TTG) inflammatory makers, leukocyte *MMP12* vs. TTG serum concentrations, MMP-9 vs. total cholesterol, (b) leukocyte *IL-6* expression vs. GGTP serum levels and serum leptin levels vs. ALT concentrations. These observations suggest in the early stages of obesity (in children, adolescents) the components of the MMP/TIMP axis may control (here down regulate) the circulating levels of total cholesterol, ALT, AST and TTG. However, leukocyte *MMP-9* expression tended to be elevated (*p* = 0.054) and plasma MMP-9/TIMP-1 ratio was significantly increased indicating that at least MMP-9/TIMP-1 axis remains increased. These findings partially confirm previous reports on increased levels of circulating MMP-9 and TIMP-1 in the obese children with hypertension [25] as well as in the adults with cardiovascular diseases characterized by elevation of MMP/TIMP system components, especially MMP-2 [26,27]. However, except for negative correlation between plasma MMP-9 and total cholesterol there were no other associations between the expression of MMP9/TIMP-1 axis and the obesity phenotype parameters. It may suggest that MMP 9 is blocked by TIMP-1 and exerts limited inflammatory activities. MMP-9 (gelatinase B) is produced by immune cells (neutrophils, monocytes) and fibroblasts and plays a crucial role in ECM remodeling. We previously reported a significant increase in leukocyte *MMP-2* expression in children/adolescents with essential hypertension [28], and up-regulation of MMP-2 expression has been found to be associated with the adipose tissue development [29]. We can speculate that increased serum MMP-9 observed in the obese children may originate from fat macrophages which display rather limited inflammatory phenotype further confirmed here by the low expression of the leukocyte *MMP-2*, decreased MMP-2/TIMP-2 ratio, low TNF levels and negative correlations between leukocyte *TIMP-2* expression and AST, ALT, and TGG plasma levels, as well as between leukocyte *MMP-12* expression and GGTP plasma concentrations. It’s possible that macrophages infiltrating the fat tissue of children with simple obesity are polarized into anti-inflammatory M2 phenotype [30]. In contrast, the fat tissue of the adult obese subjects is infiltrated by pro-inflammatory macrophages expressing M1 phenotype contributing to obesity-induce adipose tissue inflammation and insulin resistance [31]. Dying adipocytes in expanding fat tissue are surrounded by M1 macrophages visible as crown-like structures. Increasing numbers of M1 macrophages as well as activated adipocytes present in inflamed fat tissue produce TNF-α, IL-6 [32,33] and other inflammatory mediators including leptin and resistin that promote the occurrence of cardio-metabolic syndrome and hypertension in obesity [34]. Opinions about TNF-alpha are divided, as Russell et al. found that obese adolescent girls have an increased level of TNF-α receptors, which would enable TNF-α to bind more easily to its receptor and have a greater effect (such as IL-6 stimulation) without the need to increase the circulating TNF-α concentration [35]. TIMP-2 is ubiquitously, constitutively expressed protein that acts as a highly effective inhibitor of multiple MMPs including MMP-2 and MMP-9 [36]. TIMP-2 is also highly expressed in leukocytes [37]. Inflammatory process involves recruitment of systemic leukocytes into tissues, including fat tissue and liver. Nonalcoholic fatty liver disease (NAFLD), often present in the obese patients, is associated with increased serum concentrations of MMP-2, MMP-9, TIMP-1, 2, TGF-beta concentrations. Expression of these inflammatory markers is more or less related to liver fibrosis [38,39]. Also TIMP-1 and TIMP-2 liver concentrations are significantly elevated in patients with severe fibrosis as compared to those with mild or no fibrosis [40]. However, the AST and ALT levels were not increased in our study group of obese children, and mentioned above negative correlations between leukocyte *TIMP-2* and AST and ALT levels as well as inverse correlation between leukocyte *IL-6* and GGTP concentrations may suggest that M2 macrophages expressing TIMP-2 or IL-6 may prevail in the liver and fat tissue of obese children. Their action may result in a relatively low inflammatory status of both the liver and adipose tissue. This may be further confirmed by unchanged serum levels of resistin and decreased serum TNF-alpha concentrations. Resistin is produced mainly by macrophages, monocytes and pro-adipocytes and is known to be engaged in induction of insulin resistance and inflammatory responses [41]. Fat macrophages produce IL-6 which may up-regulate expression of IL-4 receptor on target cells and enhance transition of leukocytes into M2 phenotype via action of IL-4 [42]. This mechanism may down regulate GGTP expression in the liver of obese patients and limit its production. Similarly, negative correlation between leukocyte *MMP-12* and TTG serum levels suggests possible role of leukocyte MMP-12 in fat tissue ECM degradation that may protect from fibrosis and fat expansion. MMP-12 is primarily secreted by activated macrophages and exhibits high specificity for elastin degradation. Recent observations suggest that that increased expression of *MMP-12* by M2 macrophages in adipose tissue of obese subjects may be a compensatory mechanism to restrain adipose tissue expansion and insulin resistance [36]. Found here increased plasma leptin level and its strong positive correlation with liver inflammatory marker, GGTP (r = 0.964) suggest that leptin may induce pro-inflammatory reactions reflected by hepatocyte as an element of progressive, but still discrete inflammatory response in children with simple obesity. Leptin levels increase along with fat mass and is up-regulated in adipose tissue from morbidly obese patients; its expression in fat tissue and serum levels correlate with insulin resistance and body mass index [43,44].

### Strengths and Limitations

Boys were selected for the study because the hormonal activity of puberty leads not only to the development of secondary sexual characteristics and reproductive abilities, but also to significant consequences in the neuroendocrine system, and also affects the growth process and the chemical composition of the children’s bodies. In the case of boys, we eliminate hormonal changes that occur in girls, called the menstrual cycle. In girls, it is due to the cyclic maturation of ovarian follicles, which leads to repeated changes in the concentrations of sex hormones, especially estradiol and progesterone, which are the cause of cyclic changes in the concentration of gonadotropins, which is not observed in boys, and which do not affect the determination of metalloproteinases and their tissue inhibitors.

## 5. Conclusions

In children with simple obesity, very low grade inflammatory reactions prevail, reflected here by increase in the leukocyte *MMP*s inhibitor *TIMP-1*, decrease in the expression level of *MMP-2* and plasma MMP-2 as well as decline in MMP2/TIMP-2, low TNF-alpha levels and negative correlations between the expression of *TIMP-2* in leukocyte and liver (AST, ALT) or fat (TTG) inflammatory markers.

## Figures and Tables

**Table 1:** Anthropometric and clinical characteristics of the obese children and control group.

	OBESITY	CONTROL	*p* Value
No of boys	26	19	
Age (years)	12.3 ± 2.6	13.8 ± 3.1	0.077
Height (cm)	155 ± 15	168 ± 21	0.029
Weight (kg)	70.8 ± 19.9	61 ± 20	0.023
BMI (kg/m^2^)	28.9 ± 4.9	20.9 ± 3.3	0.0001
SDS-BMI	2.5 ± 1.1	0.37 ± 0.74	0.0001
ALT (IU/L)	18.3 ± 6.9	20.3 ± 6.7	0.15
AST (IU/L)	20.6 ± 4.3	22.1 ± 6.6	0.078
GGTP (IU/L)	16.6 ± 2.3	21.6 ± 4.2	0.007
Cholesterol (mg/dL)	165 ± 34	177 ± 36	0.012
TTG (mg/dL)	88.1 ± 44.6	95 ± 45.5	0.063
HDL (mg/dL)	51.6 ± 28.4	43.5 ± 8.9	0.595
LDL (mg/dL)	95.1 ± 32.6	116 ± 36	0.12
hsCRP (mg/dL)	0.22 ± 0.20	0.17 ± 0.08	0.59

ALT, Alanine aminotransferase; AST, Aspartate aminotransferase; GGTP, Gamma-glutamylotransferase; TTG, Triglicerydes; LDL, Low Density Lipoprotein; HDL, High Density Lipoprotein; hsCRP, high sensitivity C-reactive protein.

**Table 2:** Primer sequences of target genes and reference gene for SYBR Green real time PCR.

Gene	Forward Primer	Reverse Primer
*MMP-2*	TGA TCT TGA CCA GAA TAC CAT CGA	GGC TTG CGA GGG AAG AAG TT
*MMP-9*	CAA CAT CAC CTA TTG GAT CC	CGG GTG TAG AGT CTC TCG CT
*MMP-12*	TTC CCC TGA ACA GCT CTA CAA GCC TGG AAA	GAT CCA GGT CCA AAA GCA TGG GCT AGG ATT
*MMP-14*	CGC TAC GCC ATC CAG GGT CTC AAA	CGC TAC GCC ATC CAG GGT CTC AAA
*TIMP-1*	CTT CTG GCA TCC TGT TGT TG	AGA AGG CCG TCT GTG GGT
*TIMP-2*	CGA CAT TTA TGG CAA CCC TAT CA	CAG GCC CTT TGA ACA TCT TTA TCT
*IL-6*	TGA AAG CAG CAA AGA GGC ACT	GGC AAG TCT CCT CAT TGA ATC C
*G3PDH*	GCG GGG CTC TCC AGA ACA TCA T	CCA GCC CCA GCG TCA AAG GTG

MMP-2, -9, -12, -14 indicate metalloproteinase -2, -9, -12 or -14, respectively; TIMP-1, -2, tissue inhibitor of metalloproteinase -1 or -2; IL-6 Interleukin 6; G3PDH, Glyceraldehyde 3 phosphate dehydrogenase.

**Table 3:** Comparison of target gene expression patterns between obese patients and controls children. Values are median and interquartile 25–75 range.

	Obesity Patients (*n* = 26)	Control (*n* = 19)	*p* Value
*MMP-9*	2.35 (0.58–7.43)	1.00 (0.94–1.05)	0.054
*TIMP-1*	1.38 (0.67–2.04)	1.00 (0.98–1.04)	0.048
*MMP-2*	0.25 (0.10–0.69)	1.01 (0.98–1.03)	0.002
*TIMP-2*	1.06 (0.76–1.55)	1.00 (0.97–1.04)	0.645
*MMP-12*	1.29 (0.80–1.95)	1.00 (0.97–1.02)	0.368
*MMP-14*	1.34 (0.65–2.69)	1.00 (0.97–1.04)	0.372
*IL-6*	1.07 (0.57–2.01)	1.02 (0.94–1.04)	0.434

MMP-2; -9; -12; -14, metalloproteinase-2, -9, -12, -14 respectively; TIMP-1; -2, tissue inhibitor of matrix metalloproteinase-1 or -2; IL-6, Interleukin 6.

**Table 4:** Concentration of metalloproteinases and their tissue inhibitors as well as cytokines and adipokines in plasma in obese children and the control group. * Statistically significant at ≤0.05 in the Mann-Whitney U test.

Children Group	MMP-9 ng/mL	TIMP-1 ng/mL	IL-1β pg/mL	IL-6 pg/mL	TNFα pg/mL	Leptin ng/mL	Resistin ng/mL	MMP2/ TIMP2 ng/mL	MMP9/ TIMP1 ng/mL
Obesity [*n* = 26)	47.3 ± 21.3 *	110.8 ± 71.3	7.4 ± 7.0	7.6 ± 4.5	21.3 ± 15.6 *	13.7 ± 6.5 *	3.85 ± 1.2	90.8 ± 29.7 *	5.2 ± 3.7 *
Control [*n* = 23]	39.5 ± 17.3	98.7± 53.6	6.1± 2.7	8.3 ± 4.2	32.9 ± 9.25	2.4 ± 1.7	3.78 ± 1.3	122.3 ± 43.3	3.0 ± 1.7

## Data Availability

The data are available upon request.
